# Data for training and testing radiation detection algorithms in an urban environment

**DOI:** 10.1038/s41597-020-00672-2

**Published:** 2020-10-05

**Authors:** James M. Ghawaly, Andrew D. Nicholson, Douglas E. Peplow, Christine M. Anderson-Cook, Kary L. Myers, Daniel E. Archer, Michael J. Willis, Brian J. Quiter

**Affiliations:** 1 Oak Ridge National Laboratory, Oak Ridge, Tennessee 37831 USA; 2 Los Alamos National Laboratory, Los Alamos, New Mexico 87545 USA; 3 Lawrence Berkeley National Laboratory, Berkeley, California 94720 USA

**Keywords:** Experimental nuclear physics, Characterization and analytical techniques, Scientific data

## Abstract

The detection, identification, and localization of illicit nuclear materials in urban environments is of utmost importance for national security. Most often, the process of performing these operations consists of a team of trained individuals equipped with radiation detection devices that have built-in algorithms to alert the user to the presence nuclear material and, if possible, to identify the type of nuclear material present. To encourage the development of new detection, radioisotope identification, and source localization algorithms, a dataset consisting of realistic Monte Carlo–simulated radiation detection data from a 2 in. × 4 in. × 16 in. NaI(Tl) scintillation detector moving through a simulated urban environment based on Knoxville, Tennessee, was developed and made public in the form of a Topcoder competition. The methodology used to create this dataset has been verified using experimental data collected at the Fort Indiantown Gap National Guard facility. Realistic signals from special nuclear material and industrial and medical sources are included in the data for developing and testing algorithms in a dynamic real-world background.

## Abstract (summary)

**Table:** 

Measurement(s)	gamma ray photon detection events • radiation detection data
Technology Type(s)	Monte Carlo particle transport model • computational modeling technique
Sample Characteristic - Environment	city
Sample Characteristic - Location	State of Tennessee

Machine-accessible metadata file describing the reported data: 10.6084/m9.figshare.12654065

## Background & Summary

The US government performs radiation detection, identification, and localization campaigns for a variety of scenarios, including emergency response, large public gatherings (e.g., concerts and sporting events), and political events (e.g., presidential inaugurations). These radiation detection campaigns are generally conducted by trained teams equipped with radiation detection systems that can be carried by hand, mounted on an automobile, or mounted on unmanned robotic systems. The NaI(Tl) scintillation detector is one of the most commonly used radiation detectors because of its high gamma ray photon detection efficiency and relatively low cost. The crystal used in these detectors can also be easily manufactured in a variety of shapes and sizes^1–3^. Two primary streams of information can be used to analyze the signal from a NaI(Tl) detector. The first is the gross count rate, which is simply the number of photons detected in the sensor divided by time. The second exploits the response of an NaI(Tl) detector, which is proportional to the energy deposited by each interacting gamma ray. From this information, a histogram of detected photon energies called the gamma ray spectrum can be created. Because gamma rays emitted by different radioisotopes exhibit characteristic, discrete energies, the gamma ray spectrum can potentially be used to identify the radioisotope(s) detected. In addition, techniques based on the gamma ray spectrum may be less sensitive to background fluctuations, enabling the detection of radionuclides in highly dynamic backgrounds.

To rapidly determine if an illicit source of radiation is present, these radiation detection systems are often equipped with automated radiation detection, radioisotope identification, and/or source localization algorithms that alert the operators to potential events that need further investigation. These algorithms can operate on the gross count rate, the gamma ray spectrum, or a combination of both data streams. One of the main challenges with the design of these algorithms is the highly dynamic nature of the background radiation environment. Naturally occurring radioactive material (NORM), which is primarily comprised of ^40^K, ^238^U, ^232^Th, and the radioactive daughter products of the latter two isotopes, is present in different natural and man-made materials at varying concentrations and relative isotopic ratios. This variation in both absolute and relative NORM concentration in different materials means that both the gross count rate and spectroscopic signal read by a detector when moving throughout a search area can change dramatically from one location to another^4–7^. This is especially true in urban environments, where the composition of buildings and their resulting radioactive signatures is varied (e.g., a granite building may be placed directly next to a concrete building)^8^. Further, radiation interacts with different materials through a variety of physical mechanisms, exacerbating the dynamics of the measured radiation background signal.

All of the aforementioned factors contributing to the dynamic background signal can cause radiation detection algorithms to produce false alarms, which take valuable time to investigate^9,10^. Further, if the false positive rate of the algorithm is too high, the operators may become complacent with regards to these alarms which reduces the probability of detecting a true alarm. In many cases, the false positive rate can be lowered by raising the detection threshold, which often comes at the cost of lowering the true positive rate. This is obviously not ideal, as not detecting a real illicit radioactive source could have catastrophic consequences. Overall, the ideal algorithm would have a very high true positive rate and a very low false positive rate. In the real world, however, a balance between true positive rate and false positive rate must be identified for the mission based on the specifics of said mission.

**Fig. 1:**
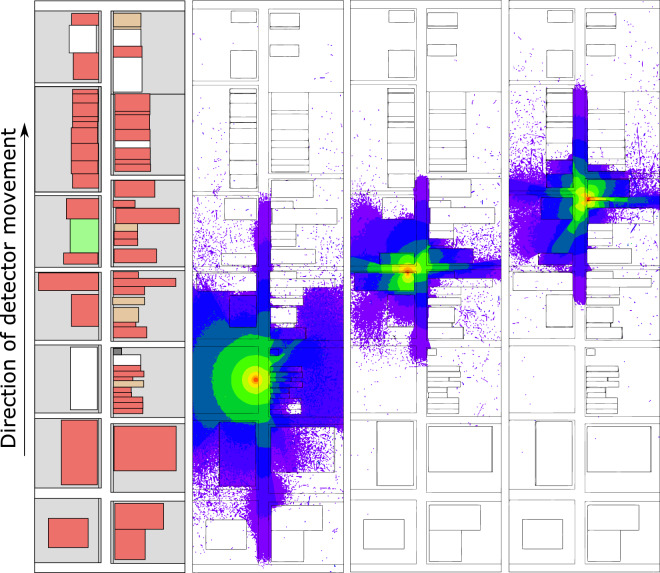
Example city street model and gamma-ray flux from three source locations. The image on the left shows an overhead view, with asphalt areas in white, concrete areas in grey, soil areas in green and buildings colored by their the type of exterior construction material (red - brick, brown - granite and dark grey - concrete). The three other images show the Monte Carlo computed flux intensity at three different source positions. Each change in color represents a decrease in flux by a factor of $\sqrt{10}$.

To provide a dataset with high quality labels to develop and evaluate radiation detection, identification, and localization algorithms, Monte Carlo particle transport models were used to simulate the response of a 2 in. × 4 in. × 16 in. NaI(Tl) gamma ray detector moving through an urban environment. Figure 1 illustrates an example output from model showing street geometry with corresponding mapping of gamma ray flux resulting from three different sources placed in different locations. The data are simulated from a simplified city street model—a street without parked cars, pedestrians, or other “clutter.” The model includes a constant search vehicle speed with no stoplights, and no vehicles arepresent around the search vehicle. The search vehicle itself is not in the model. Instead, the detector is traveling down the street alone at a vertical height of 1 m above the ground. This simple model provides a starting point for comparing detection algorithms at their most basic level. For a detailed overview on how the data were generated, see ref. ^11^.

The parameters used to develop this model are controlled and known with absolute certainty, which is an extremely difficult condition to obtain in real-world experimental data. This level of ground truth is required to accurately assess the performance of radiation detection, identification, and localization algorithms, making this dataset a valuable asset to the algorithm development community. Further, for data-driven algorithms, such as neural networks and support vector machines in the machine learning field, high-quality labels are extremely important for training these algorithms to produce the desired results. Without mislabeled data and other outliers in the training set, algorithm developers can spend less time and effort developing mitigation strategies for these problems.

This dataset was originally developed for the “Urban Nuclear Detection Challenge” data competition hosted on the Topcoder platform^12^, which was held from March through May 2019. The data package contains the data used in the competition, along with a scoring algorithm that was used during the competition to judge the performance of algorithm result entries^13^. Further descriptions of the data and scoring algorithm are presented in their respective sections. The competition results were based on the testing dataset, which was bifurcated into private scores (used for ranking and only given to the competitors at the end of the competition) and public scores (shown to everyone during the competition).

This dataset has also been used to develop and test novel data-driven radiation detection algorithms, such as the autoencoder radiation anomaly detection (ARAD) algorithm before testing them on real-world data^14^. This dataset allowed the designers of the ARAD algorithm to evaluate new algorithms on well-controlled data and develop performance metrics, such as minimum detectable activity, receiver operator characteristic curves, and probability of detection curves^14^.

## Methods

This dataset was generated using the Monte Carlo particle transport models SCALE/MAVRIC^11,15^ alongside a suite of custom automated data processing scripts written in Python. The physical models used in the Monte Carlo simulation consist of seven interchangeable city blocks, each containing a combination of buildings, sidewalks, streets, parking lots, and grassy fields. Each block is composed of some combination of asphalt, soil, concrete, granite, and brick. Each run in the dataset is a simulation of the detector response of a 2 in. × 4 in. × 16 in. NaI(Tl) gamma ray detector moving down a lane of traffic at a constant speed.

To provide a realistic background response variability in the data, the concentration of the NORM components in each material for each block was varied between different runs. In addition, instead of just one model, eight model instances were used, each with a different city block stacking configuration. To increase the difficulty to simply “learn” the geometry of each model, detector starting and ending locations are also varied and four lanes of travel are used (the detector can move in either direction through each model).

**Fig. 2:**
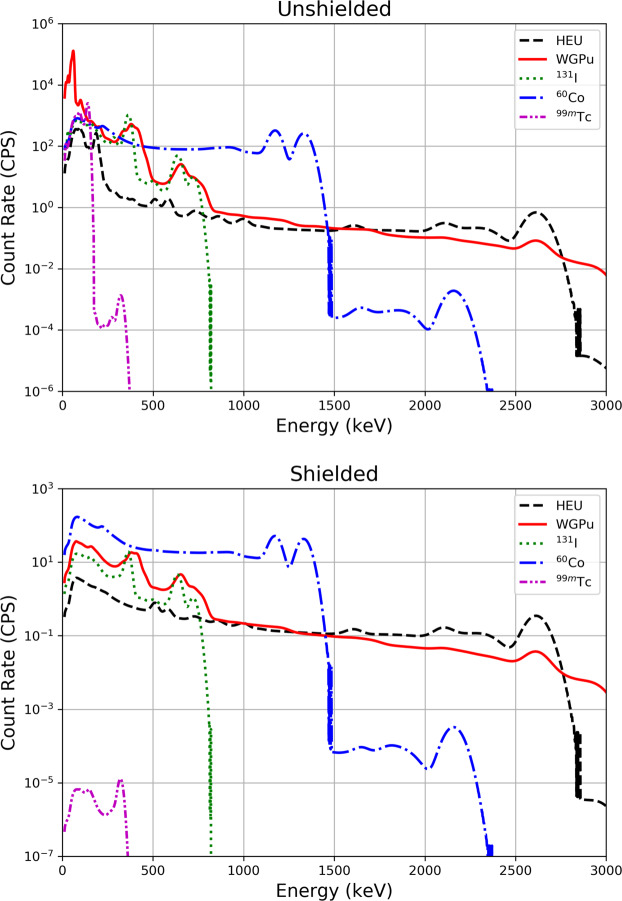
Gamma ray spectrum templates for each of the six sources modeled in the data, with and without 1 cm of lead shielding.

Six threat sources are present in some of the datasets, both with and without 1 cm of lead shielding: ^60^Co, ^99m^Tc, ^131^I, highly enriched uranium, weapons-grade plutonium, and ^99m^Tc + highly enriched uranium. The gamma ray spectral templates for both the unshielded and shielded scenarios are shown in Fig. 2. The physical locations and activity of each source varies between runs. A total of 15 source locations were used, spread among all blocks, each with a different offset from the road and varying amounts of environmental shielding. More information on the development of the Monte Carlo model and how detector response data was generated is discussed in detail in ref. ^11^.

The end result of this effort is thousands of data files resembling typical radiological search data collected on urban streets in a midsized US city. Each data file simulates the pulse trains of gamma ray detection events (amount of energy deposited in the detector at a given time) received by a standard 2 in. × 4 in. × 16 in. NaI(Tl) detector driving along several city street blocks in a search vehicle at a constant speed between 1 and 13.4 m/s. The energy resolution for this detector is 7.5 % at 661 keV. Data are divided into two categories: a training set and a test set. In the competition, the competitors were only provided answers to the training set and submitted their results online for the test set. Based on their performance, a numeric public score was generated based on a portion of the test set. A separate private score was generated on the other portion of the test set, and this score was used to rank the final submissions from each competitor.

## Data Records

The data can be found in ref. ^13^. in a 13 GB gzip compressed tarball (29 GB uncompressed). This work is licensed under CC BY 4.0. To view a copy of this license, visit https://creativecommons.org/licenses/by/4.0. To download the data, users must use either the Globus Connect Personal application to create a personal endpoint or a Globus endpoint server to download the data. Once downloaded, the tarball contains the following files:

- README.pdf—A pdf document with an overview of the dataset
- scorer—A directory containing a code to score training and test answers
- sourceInfo—A directory containing threat source templates
- submittedAnswers.csv—A template solution.csv file for the test set
- testing—A directory containing all test datasets
- training—A directory containing all training datasets
- trainingAnswers.csv—A template solution.csv file for the test set

The sourceInfo directory contains source templates for bare and shielded threat sources in the file named SourceData.csv. Plots of all source templates are also included in portable network graphics format (.png extension), as shown in Fig. 2.

The training and testing directories each contain data files in comma-separated value format (.csv extension). Each file has two columns; the first column represents the time between photon detection events in the detector in units of microseconds, and the second column is the energy of the detected photon in units of kiloelectron volts.

The scorer directory contains a Python 3 code to generate a score based on the solution templates (solution_training.csv and solution_testing.csv in the scorer directory). This code uses training and test set answers keys (answerKey_testing.csv and answerKey_training.csv) to generate training and test scores.

## Technical Validation

The accuracy of the modeling and detector response generation methodology has been studied using a radiation transport test bed of the Fort Indiantown Gap National Guard facility in Pennsylvania^16,17^. Using measurements at this facility, modeled detector response functions have been compared with experimental data for both background only and source plus background simulations^18^.

## Usage Notes

Each of the comma separated value (CSV) data files in the training and testing directories contains data in the list mode format with the first column representing the time since last detection event in units of microseconds and the second column representing the energy of the detected photon in units of keV. This data format gives the user a high level of flexibility that allows the algorithm developer to format the data into a variety of common radiation data formats. The gross count rate in the detector can be extracted from the list mode data by summing the number of total detection events (counts) over a designated period of time. Likewise, gamma ray spectra can be generated by creating a histogram of the detected photon energies over a designated time and energy window.

Another potential data format that may be useful to certain algorithms is the photon count rate in the detector for specific energy regions of interest. As stated in the Background & Summary section, gamma rays are emitted from specific radioisotopes and exhibit energy values characteristic to those isotopes. By summing the number of counts over time within a specific energy band, the count rate for only a select photon energy window can be obtained.

Some algorithms, particularly those based on neural networks designed for image analysis, can observe the gamma ray spectrum as it evolves over time in the form of an image. One way to achieve this is to stack gamma ray spectra integrated over a period of time in the form of a 3D waterfall plot. Like most image analysis tasks, normalizing the images using min–max normalization would likely be beneficial.

## Data Availability

The position- and energy-dependent flux data were generated with MAVRIC, a serial-only code in the SCALE 6.2 package^19^. Some custom codes were developed to make the mesh-based sources outside of MAVRIC, but these codes are not available. However, the methods used in these custom codes have been adopted by Shift, a new parallel Monte Carlo code, which will be released in the next version of SCALE. Shift will be able to use the same geometry and materials as MAVRIC and perform similar calculations.
